# Clinicopathological molecular characterizations of sinonasal NUT carcinoma: a report of two cases and a literature review

**DOI:** 10.3389/fonc.2023.1296862

**Published:** 2024-01-04

**Authors:** Min Chen, Shuang Li, Lili Jiang

**Affiliations:** Department of Pathology, West China Hospital, Sichuan University, Chengdu, Sichuan, China

**Keywords:** NUT carcinoma, BRD4::NUTM1 fusion, BRD3::NUTM1 fusion, next generation sequencing (NGS), sinonasal malignancies

## Abstract

**Background:**

Nuclear protein in testis (NUT) carcinoma (NC) is a rare, aggressive tumor with a typical NUTM1 gene rearrangement.

**Methods:**

Herein, we report a series of 2 cases of sinonasal NC: one in a 16-year-old woman and one in a 37-year-old man. Immunohistochemistry (IHC) staining for NUT (C52B1), fluorescence in situ hybridization (FISH), and next generation sequencing (NGS) sequencing were performed to investigate the morphological and genetic features of sinonasal NC.

**Results:**

The two cases presented similar pathological features and IHC markers, and typical morphological changes, including undifferentiated cells and abrupt keratinization, were observed, with numerous mitotic figures and widespread tumor necrosis. Diffuse expression of NUT, CK, p63, and p40 was noted, while the tumors were negative for synaptophysin, chromogranin A, S-100, EBV-ISH, and PD-L1. Both tumors harbored a NUTM1 rearrangement. Subsequent sequencing revealed a rare BRD3::NUTM1 fusion and a classic BRD4::NUTM1 fusion. In addition, MCL1 copy number gain (2.1), low tumor mutation burden and stable microsatellites, were also confirmed. Case 1 received surgery and chemoradiotherapy but died 13 months after local recurrence and subsequent lung and bone metastasis. Case 2 underwent chemoradiotherapy and unfortunately died from the disease 6 months later. A review of all previously reported cases of sinonasal NCs (n=55) revealed that these tumors occur more frequently in female pediatric patients (n=11, male: female =3:8), whereas this sex difference is not observed in adult patients (n=44, male: female =23:21). The median survival times of pediatric and adult patients were 17 and 13.8 months, respectively.

**Conclusion:**

Sinonasal NC presents typical undifferentiated or poorly differentiated cells, abrupt keratinization features and heterogeneous genotypes, including BRD4::NUTM1 and BRD3::NUTM1 fusions, with low tumor mutation burden and stable microsatellites.

## Introduction

Nuclear protein in testis carcinoma (NC) is a rare and aggressive genetically defined malignant neoplasm (1). NC was initially described in children and adolescents in 1991 (2, 3); however, the frequency of diagnoses in adults has increased in recent years. Although NC generally arises in the midline structures of the thorax or from the head and neck (HN), other sites, such as the kidney, bladder, and parotid gland, can also be involved (4). Furthermore, NC is considered the most clinically aggressive squamous carcinoma, with amedian overall survival (OS) duration of only 6.5 months; most patients succumb to rapid disease progression with early metastases to locoregional and distant sites, even with intensive treatment (4–6).

Genetically, the hallmark of NC is the t (7, 8) translocation, and this genetic aberration leads to the formation of the BRD4::NUTM1 fusion oncogene (9). The BRD-NUT oncoprotein has been demonstrated to block epithelial differentiation and maintain carcinoma cell growth. Most patients had the BRD4::NUTM1 fusion (78%); however, other fusion variants have also been described, including BRD3::NUTM1 (15%) and NSD3::NUTM1 (6%) (6). In addition, several novel fusion partner genes have identified in NUTM1-associated sarcoma (6).

NC is a poorly differentiated tumor that displays variable degrees of squamous differentiation and occasionally presents abrupt keratinization but lacks typical histological features. It is often difficult to diagnose, especially as the sinonasal tract gives rise to many tumors with undifferentiated morphologies, such as sinonasal undifferentiated carcinoma (SNUC) and poorly differentiated squamous cell carcinoma (PDSCC) (10, 11). Diagnosis is often assisted by the demonstration of NUT staining or NUTM1 rearrangement in reverse transcriptase–polymerase chain reaction (RT-PCR), fluorescence *in situ* hybridization (FISH), and next-generation sequencing (NGS) analyses (9).

To date, few cases of sinonasal NC have been reported, and its clinicopathological and molecular features have not been sufficiently clarified. Herein, we report 2 cases of sinonasal NC and their clinical and pathological presentations. We also reviewed sinonasal NC and molecular information.

## Materials and methods

### Patients and clinical samples

In this study, two sinonasal NC cases, a 16-year-old woman and a 37-year-old man were identified by NUT immunohistochemistry among 118 primary head and neck poorly squamous cell carcinomas at the West China Hospital of Sichuan University between January 2016 and December 2020, as previously described (12). All sinonasal NC tumors, including surgical resection specimens for Case 1 and endoscopic biopsies for Case 2, were sampled, fixed in 10% formalin, embedded in paraffin, stained with hematoxylin and eosin (H&E) and reviewed by two experienced pathologists (LL. J and SL).

### Immunohistochemistry and Epstein–Barr virus status analyses

A series of markers were assessed in addition to NUT (clone C52B1, CST, MA, USA), including Ki-67 (clone 9-40, Roche, AZ, USA), p40 (clone ZR8), CK5/6 (clone D5/16B4), anti-pankeratin (clone AE1/AE3), p63 (clone UMAB4), CD34 (clone EP88), synaptophysin (clone UMAB237), CD99 (clone 12E7), chromogranin A (clone LKZH10), p53 (clone D0-7), EGFR (clone EP22), and p16 (clone ICI). EBV status was assessed via EBER *in situ* hybridization (EBV-ISH) (Dako, no. Y520001). NUT protein positivity was defined as “strong” when there was speckled nuclear staining in more than 50% of the tumor nuclei (13). Positive PD-L1 expression was detected using the tumor proportion score (TPS) method (percentage of PD-L1-positive tumor cells over the total number of tumor cells on the whole entire slide) (14).

### Fluorescence *in situ* hybridization

Two sinonasal NCs were subjected to further FISH analysis using a NUTM1 break-apart probe and a BRD4::NUTM1 fusion probe. All probes were commercially purchased from Anbiping (Guangzhou, China). FISH slides were observed under a 100× objective using a fluorescence microscope (Leica DM6000, Wetzlar, Germany). Scoring was performed by two independent pathologists (MC and SL) with expertise in FISH analysis. Samples were considered FISH-positive if over 15% of the 100 scored tumor cells harbored NUTM1 break-apart or BRD4::NUTM1 fusion signals (13).

### Next generation sequencing

A targeted DNA-based NGS panel (YuanSu™ panel, Zhiben, Shanghai, China), including a 701-gene panel that included all coding exons of 638 key cancer-related genes and selected introns of 63 commonly rearranged genes in solid tumors, was performed [10]. The total DNA of the lesion and corresponding normal tissues was isolated from 5-μm-thick slices of FFPE samples using a DNA FFPE kit (Qiagen, Valencia, CA, USA). Fragments 200 to 400 base pairs in size were selected with beads and hybridized with the capture probe baits. Hybrid selection was subsequently performed with magnetic beads, and PCR amplification was carried out. The concentration of the DNA samples was determined using the Qubit 3.0 dsDNA assay (Thermo Fisher Scientific, Waltham, MA). Paired-end 2×150 bp sequencing was performed on the MiSeq DX platform (Illumina, San Diego, CA, USA) (15). The sequenced data were analyzed by STAR Fusion software (16).

## Results

### Clinical history of the two sinonasal NCs

**Figure 1:**
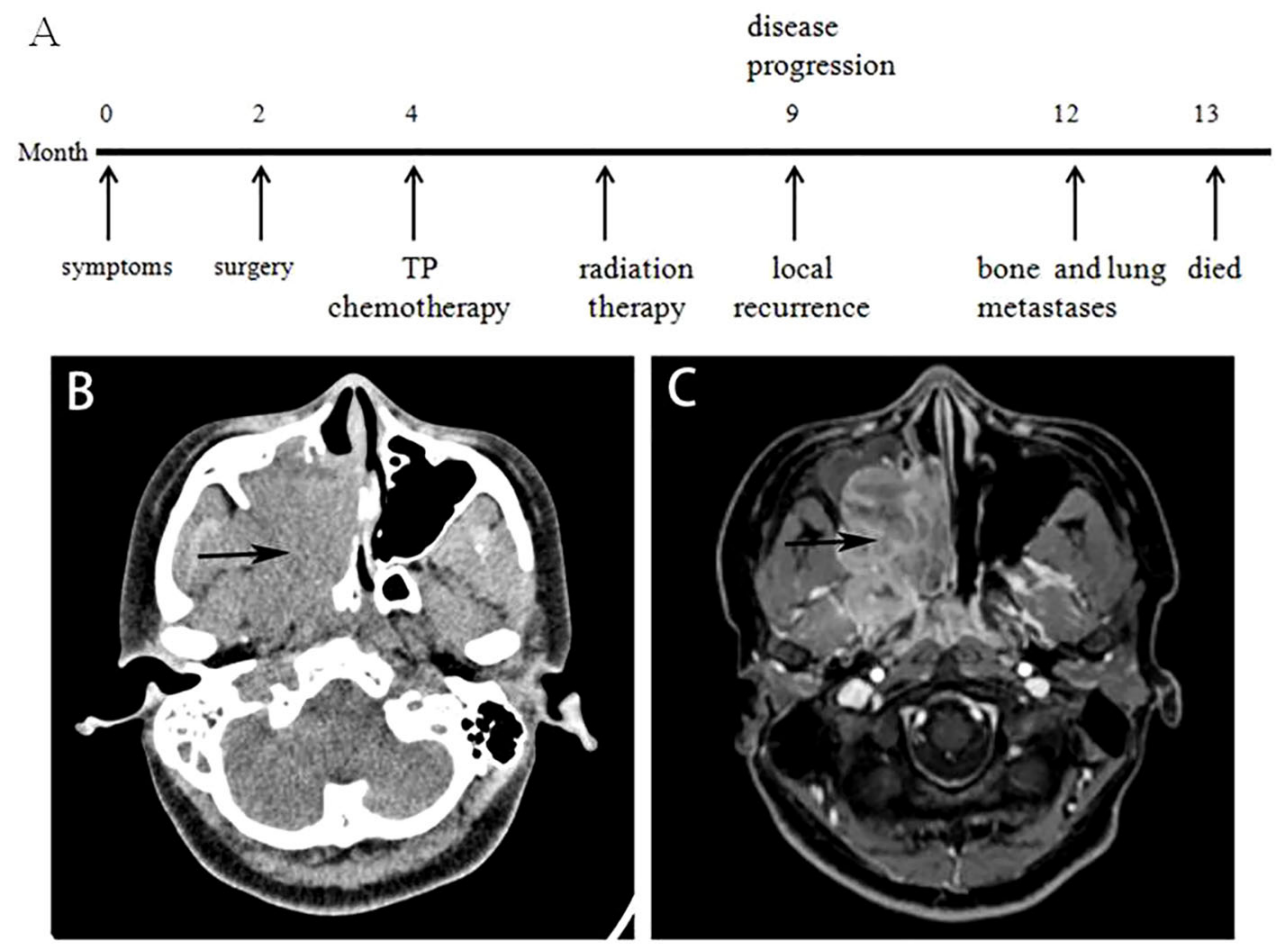
Imaging characteristics of Case 1. **(A)** Timeline of the diagnosis and treatment course of Case 1. **(B)** Contrast-enhanced CT scan of the head demonstrated a 5.1×3.1×2.3 cm sinonasal tumor filling the right maxillary sinus area and extending to the adjacent structures (black arrow). **(C)** Brain MRI with and without contrast demonstrating a sphenoid sinus mass.

Clinical and molecular information of the 2 sinonasal NCs is shown in **Table 1**. The patient in Case 1 was a 16-year-old woman with a 2-month history of right-sided nasal congestion, intractable epistaxis, tinnitus and hearing loss. When her symptoms progressed, a computed tomography (CT) head scan with contrast was performed, demonstrating a large enhancing sinonasal tumor measuring 5.1×3.1×2.3 cm that filled the right maxillary sinus area and extended to the adjacent structures (**Figure 1B**). Magnetic resonance imaging (MRI) of the brain with and without contrast demonstrated a sphenoid sinus mass (**Figure 1C**). However, no obvious abnormalities were observed in the brain. Laboratory evaluation demonstrated normal blood counts, normal renal function, uric acid and lactate dehydrogenase, mild hypercalcemia and aspartate aminotransferase elevation.

**Table 1:** Clinical and molecular information of the 2 sinonasal NC cases.

Patient	Sex/Age	Tumor location	Tumor size (cm)	Lymph node involvement	Metastasis at diagnosis	FISH NUTM1 break	FISH BRD4::NUTM1 fusion	NGS results			Treatment	Follow-up (mo)
								NUT variant	TMB (mut/mb)	Microsatellite status		
1	F/16	Right maxillary sinus	5.1×3.1×2.3	No	No	P	N	BRD3 exon 10 and NUTM1 exon 2	0.7	Stable	CRT	DOD, 13mo
2	M/37	Left nasal cavity	NA	No	No	P	P	BRD4 exon 11 and NUTM1 exon 3	0	Stable	S+CRT	DOD, 6mo

F, female; M, male; NA, not available; P, positive; N, negative; S, surgery; CRT, chemoradiotherapy; DOD, died of disease.

The patient in Case 2 was a 37-year-old man with a 1-month history of left-sided nasal obstruction, headache, purulent and bloody nasal mucus, and a decreasing sense of smell. When the patient’s symptoms worsened, he was referred to otolaryngology, where a large left nasal cavity mass was noted. Further workup, including maxillofacial and chest examination, computed tomography, and facial magnetic resonance imaging, demonstrated a large heterogeneously enhancing infiltrative mass in the left nasal cavity and sinuses. Laboratory evaluation demonstrated normal blood counts, normal hepatic and renal function, uric acid, and lactate dehydrogenase. Under nasal endoscopy, the bilateral nasal mucosa showed chronic hyperemia, and some purulent secretions were attached. The left nasal cavity was narrowed with a gray soft tumor. A small incisional biopsy revealed a malignant gray soft tumor, which visibly invaded the surrounding bone tissues. Some cancer thrombi could be seen in the vasculature.

### Histology and immunophenotypes

**Figure 2:**
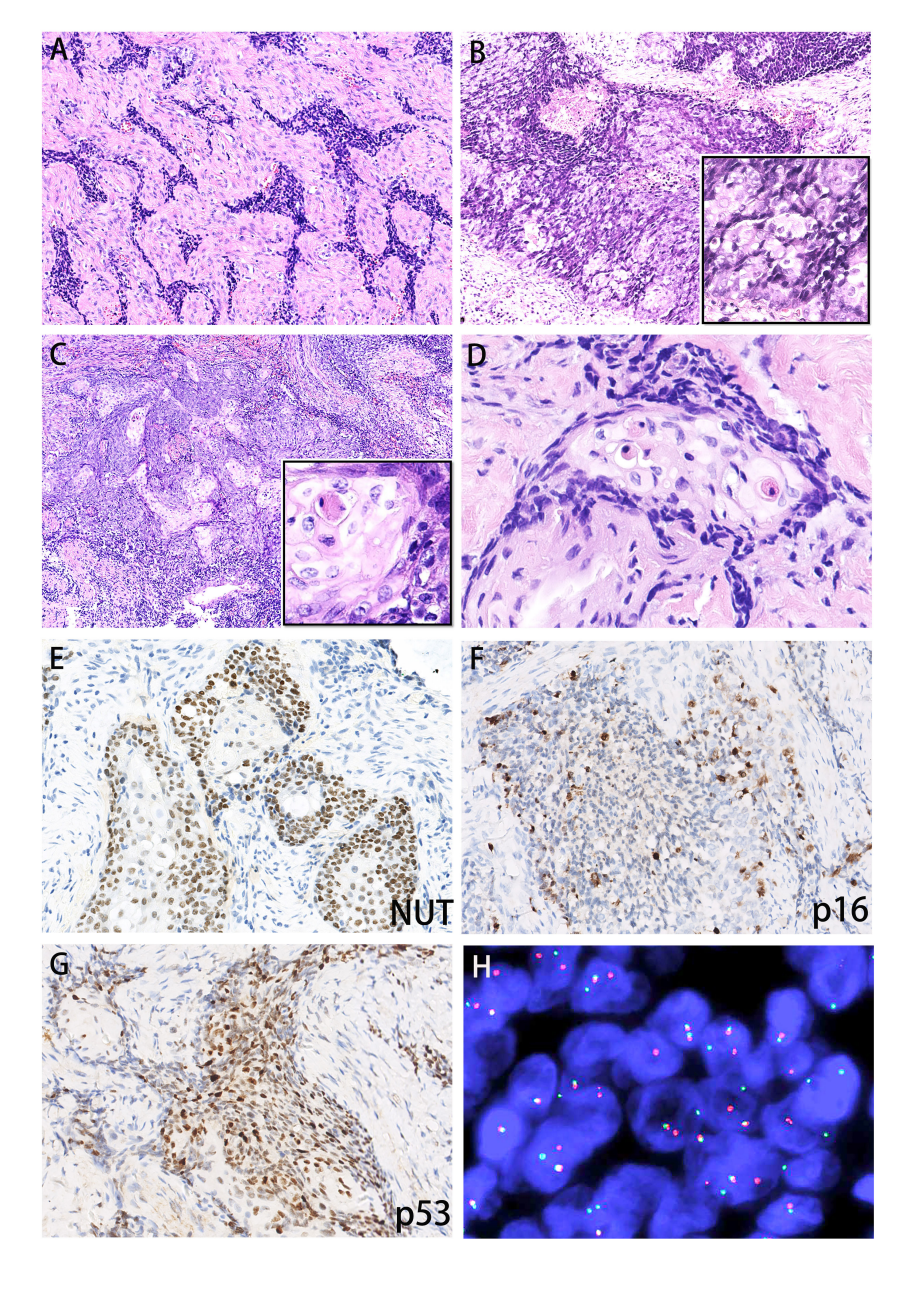
Histopathology and immunophenotypes of the two sinonasal NCs. Case 1 featured the BRD3::NUT fusion, and case 2 featured the BRD4::NUT fusion. **(A)** Poorly differentiated small squamous cells in both cases, **(B)** focal vesicular nuclei (right lower insert), **(C)** focal well differentiated small cells in Case 1 (right lower insert), **(D)** abrupt keratinization with pale eosinophilic cytoplasm, **(E)** diffuse expression of NUT was observed in both cases, **(F)** positive staining for p16 in Case 1, **(G)** positive staining for p53 in Case 1, **(H)** fluorescence *in situ* hybridization studies illustrating NUTM1 break-apart in both cases.

The lesion was composed of sheets or nests of poorly differentiated small cells, and most nuclei were round or oval-round in shape and medium in size (**Figure 2A**). In addition, vesicular nuclei were noted focally (**Figure 2B**). Moreover, focal well-differentiated cells were observed in Case 1 (**Figure 2C**). Abrupt keratinization with pale eosinophilic cytoplasm was commonly noted (**Figure 2D**). Nuclear atypia or pleomorphism was generally not prominent, but mitotic activity varied from 1/10 HPFs to 8/10 HPFs. Two tumors showed focal areas of hemorrhage and necrosis, characteristically surrounded or separated by fibrotic stroma.

Both cases showed similar immunophenotypes. The poorly differentiated tumor cells exhibited strong and uniform nuclear immunoreactivity for NUT (**Figure 2E**). The tumor cells homogenously and robustly expressed p63, CK5/6, CK7, EMA, and p40. Additionally, Case 1 showed diffuse nuclear and cytoplasmic block positivity for EGFR, p16 (**Figure 2F**) and p53 (**Figure 2G**) in well-differentiated areas. No signal was found for chromogranin A, synaptophysin, S100 or EBV-ISH in either case. PD-L1 showed weak membranous positivity in less than 1% of tumor cells. The proliferation activity as determined by the Ki67-proliferation index in hot spot areas reached 60% and 40% in Cases 1 and 2, respectively.

### Genetic results

**Figure 3:**
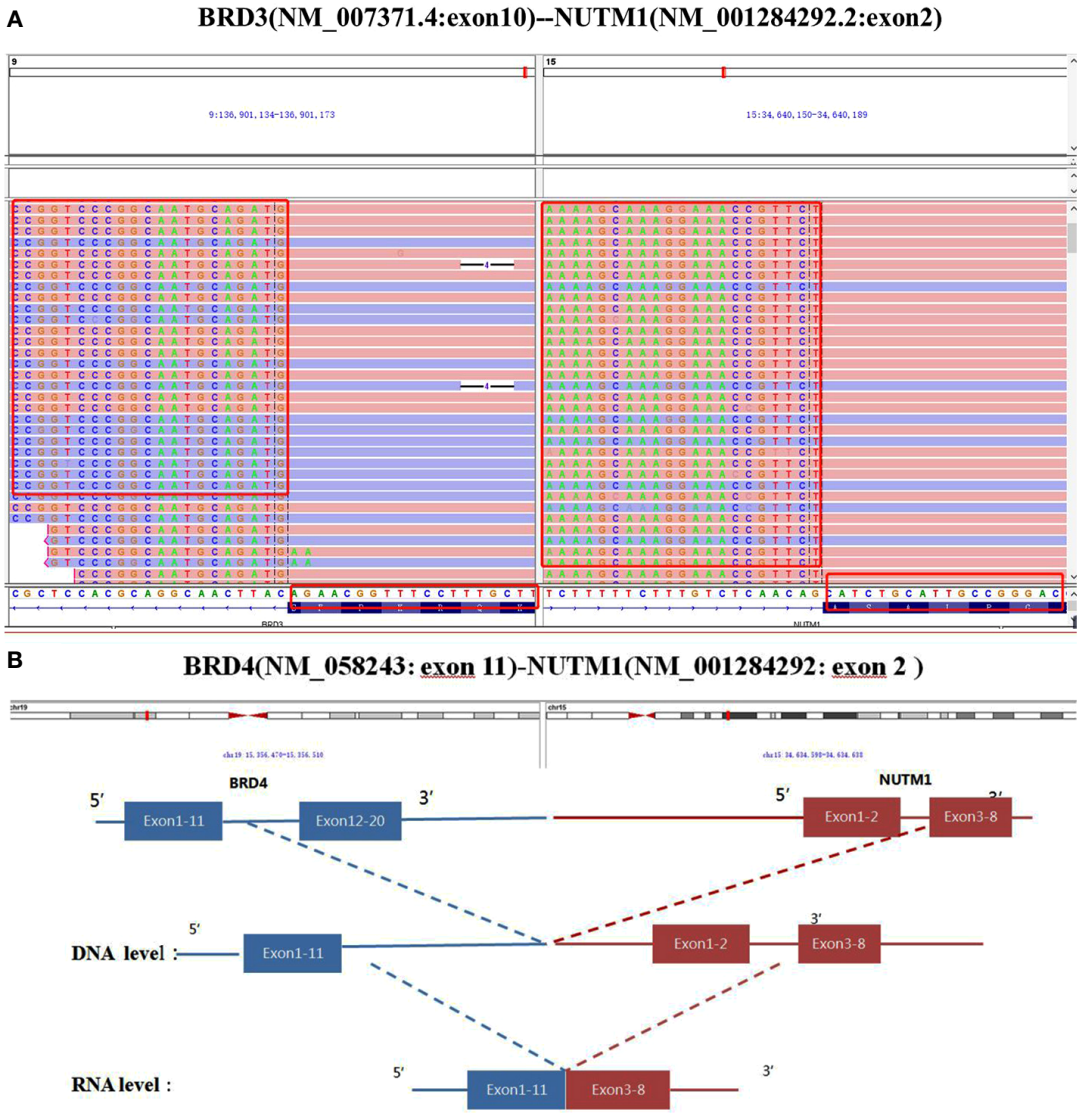
Partial nucleotide sequence of the BRD3::NUTM1 fusion transcript in Case 1 **(A)** and the BRD4::NUTM1 fusion transcript in Case 2 determined using next generation sequencing **(B)**.

In Case 1, the NUTM1 break-apart signal was observed in 84% of tumor cells (**Figure 2H**), whereas the tumor was negative for BRD4::NUTM1, SS18, and EWSR1 rearrangements. Consistent with the FISH results, Case 1 harbored a rare BRD3::NUTM1 fusion, with in-frame breakpoints in BRD3 exon 10 (NM_007371.4) and NUTM1 exon 2 (NM_001284292.2) (**Figure 3A**).

In Case 2, NUTM1 break-apart and BRD4::NUTM1 fusion signals were observed in 84% and 75% of the tumor cells, respectively, with in-frame breakpoints in BRD4 exon 11 and NUTM1 exon 3 (**Figure 3B**). Additionally, this Case showed MCL1 gene copy number gain. Both cases exhibited microsatellite stability and a low TMB, with 0.7 and 0 mutations per megabase (mut/mb) in Case 1 and 2, respectively.

### Treatment and outcomes

Two months after the surgery, the patient in Case 1 received TP chemotherapy (albumin paclitaxel-400 mg Day 1, cisplatin-40 mg, Day 1-day 2, 35 mg Day 3, intravenous glucose tolerance test, every 3 weeks). She was diagnosed with grade 4 high-frequency hearing loss as a result of cisplatin treatment. After completing 2 cycles of chemotherapy, CT/MRI revealed that the patient did not have lymph node involvement or distant metastases. She was referred for radiation therapy and subsequently developed treatment-associated radiation dermatitis of her central face. Unfortunately, local recurrence was noted within 5 months. Bone and lung metastases subsequently appeared, and the patient died 13 months later.

Despite chemotherapy with cisplatin and navelbine, the patient in Case 2 developed brain metastases and passed away 6 months after diagnosis.

### Review of studies

The Detailed clinical information about these patients is given in **Table 2**. Patient age at presentation ranged from 1 to 66 years (mean 37.9 years, median 39 years), among which 11 pediatric patients were identified (<18 years old). There were 26 males and 29 females. Interestingly, the sex predilection for pediatric sinonasal NC was prominent, with females constituting 72.3% (n=11, male: female =3:8). In contrast, the adult patients displayed an almost equal gender distribution (n=44, male: female =23:21). Furthermore, sinonasal NC tends to involve the frontal and ethmoidal sinuses more frequently than other sinonasal cancers.

**Table 2:** Clinicopathological features of 55 reported sinonasal NC cases.

Case	Year	Age	Sex	Histologic features	NUT IHC	NUT variants	Therapy	Recurrence /Metastasis	Outcome (months)	Reference
1	2004	26	M	UDC with SD	NA	BRD4::NUT	CT+RT	Bone	7	French CA et al. 1 (10)
2	2008	31	M	UDC	P	BRD4::-NUT	S+CT+RT	NA	10	Stelow EB et al. (10)
3		39	F	UDC with focal SD	P	BRD4::NUT	S+CT+RT	NA	7	
4		40	F	UDC with focal SD	P	BRD4::NUT	NA	NA	NA	
5		47	M	UDC	NA	BRD4::NUT	NA	NA	NA	
6	2011	54	F	NA	NA	BRD4::NUT	RT	Orbital ridge and forehead	7	Davis BN et al. (8)
7	2011	54	F	UDC	P	NA	CT+RT	NA	12	Hsieh MS et al. (17)
8	2012	26	M	UDC	P	NA	S+CT+RT	Bone, LN	8	Bishop JA et al. (11)
9		33	M	UDC	P	NA	S+CT+RT	Bone, LN	11	
10		48	M	Squamous cell carcinoma	P	NA	S+CT+RT	Bone	16	
11	2013	42	M	PDSC	P	BRD4::NUT	CT+RT	Intracranial	9	Fang W et al. (18)
12		50	M	UDC	P	BRD4::NUT		Intracranial	1	
13	2014	18	F	UDC without SD	P	BRD4::NUT	S+CT+RT	Ethmoid bone	NA	Suzuki S et al. (19)
14	2015	14	F	UDC with abrupt SD	P	BRD4::NUT	S+RT	No	3	Stirnweiss A et al. (20)
15	2015	26	M	UDC	P	NA	S+CT+RT	Orbital floor/left hard palate	18	Solomon LW et al. (21)
16	2016	20	M	PDC with focal SD	NA	BRD4::NUT	S+CT+RT	Cervical LN	8	Klijanienko J et al. (22)
17	2016	26	F	Undifferentiated malignant neoplasm	NA	NA	NA	NA	NA	Bishop JA et al. (23)
18		48	F	Squamous cell carcinoma	NA	NA	NA	NA	NA	
19		56	F	Atypical cells	NA	BRD4::NUT	NA	NA	NA	
20		36	F	NC	NA	BRD3::NUT	NA	NA	NA	
21	2017	30	M	PDSC	P	NA	NA	NA	NA	Kakkar A et al. (24)
22		31	F	UDC with focal SD	P	NA	S+RT	Left orbit	2	
23		25	M	UDC	P	NA	NA	NA	NA	
24		10	F	PDC with SD	P	NA	CT+RT	Anterior ethmoids/left lacrimal fossa	NA	
25		30	F	UDC	P	NA		Left orbit	NA	
26	2017	53	F	PDC with SD	P	BRD4::NUT	S+CT+RT	Local recurrence	NA	Edgar M et al. (25)
27	2017	56	F	Undifferentiated small round cell morphology without SD	P	BRD4::NUT	CT+RT	Liver, lungs, pleura, spleen, adrenal glands, LNs, and bones	10	Minato H et al. (26)
28		66	F	Undifferentiated small round cell morphology without SD	P	BRD4::NUT	CT+RT	Liver and bones	13	
29		1	M	Undifferentiated small round cell morphology without SD	P	BRD4::NUT	CT+RT	Liver and bones	15	
30	2018	49	M	NA	P	NA	CT+RT	LN	9	Arimizu K et al. (27)
31	2018	48	M	UDC with SD		BRD4::NUT	NA	Orbit	NA	Chan W et al. (28)
32	2018	60	F	Squamous cell carcinoma	P	NUT	S+RT	No	3	Laco J et al. (29)
33		65	M	Squamous cell carcinoma	P	NUT	S+RT	No	108 NED	
34		46	M	Basaloid squamous cell carcinoma	P	NUT	S+RT	No	8	
35	2019	48	M	Squamous epithelium	P	BRD4::NUT	S+CT+RT	No	6/alive	Albrecht T et al. (30)
36	2019	60	F	PDSC	P	NA	S+RT	LN	12	Lee T et al. (31)
37		45	F	PDSC	P	NA	S+CT+RT	No	36/alive	
38		42	M	PDSC	P	NA	CT	LN	8/alive	
39		29	M	PDSC	P	NA	NA	No	NA	
40	2019	8	M	PDC	P	NUT	S+CT+RT	NA	16.4	Jung M et al. (32)
41		47	M	PDC	P	NUT	S+CT+RT	NA	9.3	
42		48	F	PDC	P	NUT	S+CT+RT	NA	18.7	
43		51	M	PDC	P	NUT	S+CT+RT	NA	10.6	
44		59	F	PDC	P	NUT	CT+RT	NA	3	
45		64	M	PDC	P	NUT	S+CT+RT	NA	8.2	
46		66	F	PDC	P	NUT	NA	NA	11.4	
47	2019	9	F	Nasopharyngeal carcinoma	P	NA	CT+RT	Local recurrence	2	Prasad M et al. (33)
48		17	M	NC	P	NA	S+CT+RT	Local recurrence	10	
49		13	F	UDC	P	NA	S+RT	No	21/alive	
50	2020	12	F	UDC with SD	P	NA	S+CT+RT	No	40/alive	Sopfe J et al. (34)
51	2020	15	F	Sinonasal papillary neoplasm	P	BRD3::NUT	CT+RT	No	34/alive	Leeman R et al. (35)
52	2021	39	F	PDC	P	YAP1::NUT	S+CT+RT	Bilateral pulmonary	9	Patel SA et al. (36)
53	2021	56	F	PDC	P	NA	S+CT+RT	Vertebral and liver	6	Crocetta FM et al. (37)
54	2022	37	M	PDC with SD	P	BRD4::NUT	CT+RT	No	6	This study
55		16	F	PDC with SD	P	BRD3::NUT	S+CT+RT	Lung and bone	13	

M, male; F, female; S, surgery; CT, chemotherapy; RT, radiotherapy; OS, overall survival; IHC, immunohistochemistry; ND, not done; NA, not available; SD, squamous differentiation; PDC, poorly differentiated carcinoma; PDSC, poorly differentiated squamous carcinoma; UDC, undifferentiated carcinoma; LN, lymph node; NC, NUT carcinoma.

The most common histology of sinonasal NC was poorly differentiated or undifferentiated carcinoma (46 of 55 cases, 83%); however, abrupt keratinization was seen in 40% (22 of 55) of cases. Tumors lacking evidence of epithelial differentiation or where histologic classification was not specified composed the remaining (5 of 55, 9%) cases. This observation was consistent with previous studies. Sinonasal NC demonstrates a consistent immunoprofile similar to that seen at other anatomic sites. We found that 46 sinonasal NC cases with available data all stained positive for NUT. The tumor commonly expresses cytokeratins and squamous markers such as p63, with variable expression of p40 and CK proteins. Some reports described patchy staining of synaptophysin, p16, or even TTF-1 and tumor EBER-negative status.

## Discussion

The head and neck region is the second most common primary site of NCs, comprising 40% of all NC cases (7). Sinonasal NC is undifferentiated malignant neoplasm (1). Recently, Wang et al. reported 3 sinonasal NCs identified from 145 sinonasal malignancies (38). Ramesh et al. found 12 sinonasal NCs, which was the largest single-institutional cohort reported to date (39). In this study, we reported 2 cases of sinonasal NC and reviewed 53 other cases reported in the literature (8, 10, 11, 17–37, 40).

The frequency of the NUTM1 variant in sinonasal tissue is consistent with that observed in NC overall. Of the NUTM1-fusion-positive cases, BRD4::NUTM1 fusion accounted for 83% (19/23), BRD3::NUTM1 accounted for 13% (3/23) and one rare YAP1::NUTM1 fusion was observed (36). The genetic basis of NC progression and transformation has been studied in several cohorts using next-generation sequencing technologies (41–43). Lee et al.’s findings suggest that this single catastrophic event involving NUTM1 rearrangement in proliferating normal cells could be sufficient for neoplastic transformation into NUT carcinoma (44). Stefano et al. first reported that metastatic NC patients carried other somatic mutations, including deletions in colorectal cancer (DCC), mixed lineage leukemia protein 3, and splicing factor 3B subunit genes in NC cells (41). Moreover, the most highly and recurrently mutated genes in NC are associated with the Wnt, MAPK, and PI3K signaling pathways (42). Additional frequently mutated genes have also been discovered in NC, including mutations in MYC, p63, and MED24 (43). MYC is a master regulator of cell proliferation and metabolism and is central to the pathogenesis of many human cancers (45, 46). Various types of MYC gene mutations are present in diffuse large B-cell lymphoma (DLBCL) and show different impacts on MYC function and clinical outcomes (47). Unlike MYC gene translocations and overexpression, most MYC gene mutations may not have a role in driving lymphomagenesis (47). Previous studies demonstrated that MYC is a downstream target of BRD-NUT, and targeting MYC was necessary and sufficient for the blockade of NUT midline carcinoma differentiation (48). However, there are few data on sinonasal NC, and most studies have focused on pulmonary NC. In the present study on sinonasal NC, we identified one additional genomic alteration in myeloid cell leukemia-1 protein (Mcl-1). Mcl-1 is an antiapoptotic protein in the Bcl-2 family that is essential for the survival of multiple cell lineages and is highly amplified in human cancer (49). Yasuda Y et al. suggested that MCL1 inhibition therapy be applied for high MCL1- and low BCL-X L-expressing small-cell lung cancer patients (50), but it is unclear whether direct inhibition of MCL1 is also useful for sinonasal NC.

NC is aggressive and the majority of NC patients have regional and/or distant metastases at the time of presentation (4–6). Imaging typically reveals an extensively infiltrative tumor with frequent involvement of the orbit and cranial cavity (1). Of the 55 reviewed sinonasal NCs in this study, disease progression was evaluable for 43 patients, including 14 patients (33%) who had isolated locoregional disease, 10 (23%) who had isolated distant disease, and 2 (5%) who developed both locoregional and distant disease. The remaining 17 patients did not develop local or distant metastases. The outcomes and responses to therapy vary based on the anatomical site. Compared to the median OS time for NC of all ages and locations (4.7–6.7 months) (4) and the head and neck region overall (9.7 months) (5), sinonasal NC appears to have a better prognosis (13.8 months). Pediatric patients with sinonasal NC have the longest OS duration (17 months). Similarly, Chau NG et al. proposed a survival tree regression and identified three statistically distinct risk groups among 124 patients classified by anatomical site and genetics (6). They found that nonthoracic primary, BRD3, or NSD3::NUTM1 NC patients had longer survival durations (3 years, n=12) than nonthoracic primary, BRD4::NUTM1 NC patients. Nonthoracic primary NC with nonBRD4::NUTM1 fusion conferred the best prognosis, followed by nonthoracic primary NC with BRD4::NUTM1. However, primary thoracic NC patients had an average OS duration of only 4.4 months. In this study, the patient in Case 1 had a BRD3::NUTM1 fusion and a longer survival duration than the patient in Case 2 with the classic BRD4::NUTM1 fusion (13 months vs. 6 months), which is in accordance with the findings of previous studies.

A standard treatment strategy has not been established for NC, but surgery or radiation was reported to significantly improve survival (6, 7). Chemotherapy is generally ineffective, although successful treatment and long-term survival have rarely been reported in cases treated with ifosfamide-based regimens (36). To date, the majority of sinonasal NC patients have received intensive traditional multimodality therapy, consisting of various combinations of surgery, chemotherapy, and radiotherapy. Recently, several promising classes of agents, including bromodomain and extraterminal motif (BET) inhibitors (7, 51), histone deacetylase inhibitors, and immune checkpoint inhibitors (ICIs) (52), have emerged as candidates for the treatment of NC. Currently, there is no single biomarker that can reliably predict the response to ICIs. The expression of PD-L1 by tumor cells (TPS) has been the most widely studied. Some patients with high PD-L1 expression do not respond to ICIs, whereas a small proportion of patients with no PD-L1 expression respond to ICIs (52). We previously reported two low PD-L1 expression NCs, presenting a diverse response to immunotherapy. Patient 1 exhibited a poor response and soon showed tumor progression and metastasis; however, patient 2 responded remarkably and achieved pathologic complete response (pCR) without uncontrollable adverse events (53).

Additional biomarkers that have been shown to predict ICI treatment response include TMB and microsatellite instability. He et al. first applied whole transcriptome RNA sequencing to determine TMB and microsatellite instability in NC (54). Their data demonstrate that TMB ranges from intermediate (between 5 and 20 mut/mb) in an adult case to low (<5 mut/mb) in pediatric cases. Other studies have reported low TMB and stable microsatellites in NC (55). Riess et al. described 31 solid tumor cases harboring a BRD4::NUT translocation. The cohort was all microsatellite stable and harbored a low TMB (mean 1.7 mut/mb, range 0–4) (56). In our previous study, pulmonary NCs also had stable microsatellites and a lower TMB, ranging from 0.5 to 1.7 mut/mb(median 1.25) (12). Consistent with these studies, the sinonasal NC patients in this study presented low TMB and stable microsatellites. Although the number of cases is limited, the results suggest that therapy with ICIs may be beneficial in some patients; and should be further studied in this patient population.

This study has some limitations. First, this was a retrospective study from a single center. Second, because of the rarity of NUT carcinoma cases, the number of samples was too small, and only two patients were included. Finally, because of insufficient tumor samples, we did not investigate the tumor immune microenvironment in the two patients.

In conclusion, sinonasal NC presents typical undifferentiated or poorly differentiated cells, abrupt keratinization features and heterogeneous genotypes, including BRD4::NUTM1 and BRD3::NUTM1 fusions, with low tumor mutation burden and stable microsatellites.

## Data availability statement

The datasets presented in this study can be found in online repositories. The names of the repository/repositories and accession number(s) can be found below: DNA Data Bank of Japan (DDBJ) and BioSample accession(s): SAMD00665363, SAMD00665364.

## Ethics statement

The studies involving humans were approved by West China hospital, Sichuan University. The studies were conducted in accordance with the local legislation and institutional requirements. The participants provided their written informed consent to participate in this study. Written informed consent was obtained from the individual(s) for the publication of any potentially identifiable images or data included in this article.

## Author contributions

MC: Data curation, Writing – original draft. SL: Formal Analysis, Writing – original draft. LJ: Conceptualization, Writing – review & editing.
